# How to distinguish medicalization from over-medicalization?

**DOI:** 10.1007/s11019-018-9850-1

**Published:** 2018-06-27

**Authors:** Emilia Kaczmarek

**Affiliations:** Ethics Department, Center for Bioethics and Biolaw, Institute of Philosophy, University of Warsaw, ul. Krakowskie Przedmieście 3, 00-097 Warsaw, Poland

**Keywords:** Medicalization, Over-medicalization, Boundaries of medicine, Moral evaluation of medicalization, Guiding questions, Pragmatic approach

## Abstract

Is medicalization always harmful? When does medicine overstep its proper boundaries? The aim of this article is to outline the pragmatic criteria for distinguishing between medicalization and over-medicalization. The consequences of considering a phenomenon to be a medical problem may take radically different forms depending on whether the problem in question is correctly or incorrectly perceived as a medical issue. Neither indiscriminate acceptance of medicalization of subsequent areas of human existence, nor criticizing new medicalization cases just because they are medicalization can be justified. The article: (i) identifies various consequences of both well-founded medicalization and over-medicalization; (ii) demonstrates that the issue of defining appropriate limits of medicine cannot be solved by creating an optimum model of health; (iii) proposes four guiding questions to help distinguish medicalization from over-medicalization. The article should foster a normative analysis of the phenomenon of medicalization and contribute to the bioethical reflection on the boundaries of medicine.

## Introduction

For the purposes of this article, I use the following sociological definition of medicalization, according to which X is medicalized when it “is defined in medical terms, described using medical language, understood through the adoption of a medical framework, or ‘treated’ with medical intervention” (Conrad 2007, p. 5). Thus, medicalization consists in interpreting newer and newer aspects of reality,^1^ including human behaviour, in medical terms, and treating them as medical problems rather than e.g. social, political or existential ones. The history of the notion of medicalization clearly indicates that from its very beginning it has been associated with the criticism of expansion of medicine and perceiving it as an instrument of social control (Zola 1972; Szasz 1970, 2007; Illich 1976, 2010; Busfield 2017). Over time, however, some sociologists and bioethicists (Broom and Woodward 1996; Parens 2013) started to appreciate positive aspects of medicalization as well. Therefore, how should we assess this phenomenon?

The aim of this paper is to outline the pragmatic criteria for distinguishing between medicalization and over-medicalization. The article: (i) identifies various consequences of both well-founded medicalization and over-medicalization (Table 1); (ii) demonstrates that the issue of defining appropriate limits of medicine cannot be solved by creating an optimum model of health; (iii) proposes four guiding questions to help distinguish medicalization from over-medicalization and to analyse specific examples of medicalization (Table 2). The article should foster a normative analysis of the phenomenon of medicalization and contribute to the bioethical reflection on the boundaries of medicine.

## Various effects of medicalization and over-medicalization

According to Erik Parens, medicalization is wrong “when the institution of medicine oversteps its proper limits” (Parens 2013). In order to discuss over-medicalization of a phenomenon, the latter must be demonstrated to have been wrongly recognised as a medical problem, whereas in fact it is e.g. a political or a cultural one—or it has been simply misinterpreted as a problem in the first place. Furthermore, inadequate identification of the underlying cause of a phenomenon results in taking inadequate measures aimed to eliminate it. As Parens wrote:

… as medicine focuses on changing individuals’ bodies to reduce suffering, its increasing influence steals attention and resources away from changing the social structures and expectations that can produce such suffering in the first place. The idea is that, for example, rather than changing the bodies of shy people with drugs, we could change our expectations of how people behave in novel situations; again, doing so, would exemplify the virtue of learning to affirm natural variation. Further, changing social expectations would be fairer to individuals, who, instead of changing their bodies to better fit dominant norms, could, again, be affirmed in their norm challenging variation (Parens 2013, p. 30).

Then, in the context of over-medicalization, inadequate response to a problem means, first and foremost, unnecessary clinical interventions, which always entail certain health risks. However, medicalization also affects other aspects of life of individuals and communities that are not directly related to health. The table below illustrates selected opportunities and risks that may be produced by medicalization processes in various domains of individual and collective life:

**Table 1:** Medicalization impact assessment—own elaboration

	Over-medicalization-risks	Well-founded medicalization—opportunities
Health effects	Harm to health caused by undue treatment (overdiagnosis, overprescription), iatrogenic diseases, health risk related to medical procedures, undesirable side effects of the medication administered	Possibility of using tools of evidence-based medicine, e.g. treating acute mental disorders at a psychiatric hospital instead of undergoing an exorcism
Economic effects	Suboptimal expenditure and waste of public or private money, e.g. costs of treatment of iatrogenic diseases and consequences of medication errors	Improvement in the financial situation of individuals whose condition has officially been recognised as a disease, e.g. through granting insurance coverage, reimbursement of medicines, entitlement to take a sick leave
Psychological effects	Stigmatising certain conditions, individuals or their behaviour as sick; restriction of personal freedom; pressure to adjust one’s own needs and behaviour to fit the prevailing standards, e.g. pharmacological treatment of low sexual desire in women	De-tabooisation of disease, explanatory value: patients gain the possibility to understand the causes of their condition and see that they are not the only ones to suffer from it
Social effects	Ignoring social, political and interpersonal background of certain phenomena and inadequate reactions stemming therefrom, such as treating victim’s masochistic personality disorder as the cause behind domestic violence	Raising health awareness of the public, recognising medical grounds for particular behaviours and starting treatment instead of punishing the patient, e.g. limited criminal liability of the mentally handicapped persons

As shown in the Table, the consequences of considering a phenomenon to be a medical problem may take radically different forms depending on whether the problem in question is correctly or incorrectly perceived as a medical issue. Neither indiscriminate acceptance of medicalization of subsequent areas of human existence, nor criticising new medicalization cases just because they are medicalization can be then justified. Based on what criteria, however, can we establish when medicine oversteps its boundaries?

## Medicalization and models of health

As many authors (Parens 2013; Vogt et al. 2016; Saracci 1997) have rightly pointed out, the broad, holistic definition of health makes it more difficult to criticise over-medicalization. If, as defined by the World Health Organization, health is “a state of complete, physical, mental, and social well-being”, then every aspect of individual and collective life can be seen as a health problem. The broad definition of health requires widening objectives of medicine. In this model of health, medicine becomes a domain with a potential to give individuals not only *healthy*, but also *good* lives, that it provides an adequate tool for humanity to pursue perfect happiness. This problem has been discussed as follows by Daniel Callahan:

association of health and general well-being as a positive ideal, has given rise to a variety of evils. Among them are the cultural tendency to define all social problems, from war to crime in the streets, as “health” problems; the blurring of lines of responsibility between and among the professions, and between the medical profession and the political order; the implicit denial of human freedom which results when failures to achieve social well-being are defined as forms of “sickness,” somehow to be treated by medical means (1973, p. 78).

The criticism of the broad and value-laden concept of health is paradoxical due to the fact that it emerged in opposition to the biomedical model of health and disease (Boorse 1975), characterised by reductionism (treating the disease rather than the patient) and recognition of the category of disease as morally and politically neutral, which were supposed to be among the reasons for excessive dominance of medicine (Doust et al. 2017a, b). Thus, the broad concept of health emerged, among others, from the criticism of medicalization^2^ (Boddington and Räisänen 2009; Schramme and Edwards 2017), and then itself was accused of fostering medicalization:

Around the middle of the twentieth century there was increasing dissatisfaction with the dominant model of health offered by biomedicine. The preoccupation with disease and illness made it less able to deal with any positive concept of health. The ideology which viewed the individual in mechanistic ways justified ever-increasing use of medical technologies. (…) The concept of social or holistic health (…) is a different construction, locating biological processes within their social contexts, and considering the person as a whole rather than a series of distinct bodily systems. (…) In 1948 the World Health Organization defined health as ‘a state of complete physical, mental and social well-being, and not merely the absence of disease or infirmity’, and this is generally held to epitomize the social model of health. There are obvious problems about this definition, which seems to incorporate the whole of human existence (Blaxter 2010, pp. 16–19).

However, none of the models of health proposed in the history of philosophy or sociology is free from theoretical and practical problems, nor does it guarantee protection against over-medicalization. These models include concepts such as: health as absence of disease; health as the norm, typical state of the body; health as harmony, homeostasis, balance of the organism; health as the ability to function according to one’s own goals and duties; finally, health as a subjective or objective phenomenon.

Accordingly, diverse phenomena have been blamed to be the factors contributing to over-medicalization, some deriving from the biomedical model, such as: extension of official classifications of diseases—ICD-10, DSM-IV (Sedler 2016) and overdemanding health-related norms and standards, quite different depending on the scientific society that has established them,^3^ or self-tracking practices, that is to say monitoring the state of one’s own body (e.g. blood pressure, pulse, calories burned, blood sugar) using mobile devices and applications (Gimbert and Lapointe 2015), as well as Eastern medicine, based on the holistic idea of internal harmony (Hsu 2013, p. 197), or finally treating medicine as a tool of wish-fulfilling and helping patients improve their functioning or accomplish their life goals (Pellegrino 2004; Maturo 2013).

Thus, the history of the notion of medicalization reveals certain ambivalence. On the one hand, criticism concerned alleged neutrality and objectivity of medical practices (Foucault 1973; Szasz 1970), and on the other—criticism concerned invention of non-existent diseases and marketing-created health needs (Moynihan et al. 2002), which refers us back to the criterion for distinguishing real, objective needs from those artificially created. Moreover, the pursuit of universality and objectivity is an inherent part of the logic and ethos of all scientific practice (Merton 1973), including medicine. And yet, that nature has indeed provided us with no neutral definition of norm or any precise method to tell between the healthy and the sick in every situation. We will always come across phenomena that require interpretation. As Parens puts it: “the ethical responsibility for deciding whether or not to intervene falls to us and our value-laden interpretations of nature; we can’t rely on the hoped-for, value-free guidance from nature” (2013, p. 31).^4^

So how can well-grounded medicalization be distinguished from over-medicalization, if none of the models of health and disease can eliminate the risk of over-medicalization?

## Over-medicalization: a pragmatic approach

It is likely that establishing simple and clear-cut criteria for drawing appropriate boundaries of medicine is not possible (Murano 2017). Medicine is a social practice used to cope with certain problems—it provides us with an interpretative framework and methods to address those issues. Therefore, in order to assess medicalization in specific cases, we need to compare and contrast different interpretative frameworks (Rose 2007a, p. 702).

In secularised Western societies, medicine has in many aspects substituted religious institutions: the authority of a therapist has replaced that of a priest, and deviation started to be perceived in terms of sickness rather than sin or crime (Turner 1995; Rosecrance 1985; Bull 1990). But the fact of religious institutions being supplanted by medical institutions cannot be readily classified as a negative or positive phenomenon. Some critics of medicalized death believe^5^ that we ought to go back to the times when forthcoming death used to be an essentially religious experience, not a medical one, arguing in favour of dying at home and shifting the duty of caring for those on their deathbed from public and medical institutions back onto the family and religious community. However, the change in the conditions of dying has not been forced on societies—it originated from transformations that these societies have undergone, from evolving needs and priorities (e.g. striving to postpone the moment of death, to eliminate physical pain). Therefore, in order to effectively criticise the medicalization of a problem, one needs to find an alternative explanation and a solution that would be more adequate and helpful in a given situation.

For instance, according to the aforementioned Parens (2013), treatment of a woman suffering due to being stuck in relationship with a partner addicted to alcohol should not consist in diagnosing her with depression and administering antidepressants to help her cope with the situation, even if this would considerably minimise her subjective suffering. The cause of the problem here is not at the molecular level, the distress doesn’t stem from here, but rather it derives from external, objective and interpersonal factors. An adequate solution to the problem would be to sever the ties that bind her to her partner until he recovers from alcoholism. Nonetheless, Parens does not voice any concerns about medicalizing the addicted partner. This is not because we have access to any ‘nature’ or ‘essence’ of alcoholism which would prove that it is a medical problem rather than an existential or social one, but simply due to the fact that the well-established means of combating alcoholism, including various forms of therapy and pharmaceuticals affecting the metabolism of alcohol, have proved helpful for many, and no alternative, more effective response to the problem is in sight.

How can this pragmatic approach to medicalization be further developed? The idea is to shift the attention from the questions “What is a disease?” or “How should it be defined” to the questions “What should medicine—as a social practice—be concerned with?” and “How should we assess specific cases of medicalization?”.^6^ A series of guiding questions is included below to facilitate the distinction between medicalization and over-medicalization.

Whenever X (where X can be a phenomenon, a state of the body, a behaviour or a sensation) starts to be considered a medical problem, the following questions should be asked:

1. Has X been rightly recognised as a problem?
  Does X cause or significantly increase the risk of considerable physical or mental discomfort, suffering, impairments or death?
2. Does recognising X as a problem not result from unfounded, exaggerated social expectations?
  Is recognising X as a problem not an example of undue limitation of diversity of individuals for the sake of normalisation?

If we decide that a phenomenon, state of the body, behaviour or feeling is rightly considered to be a problem, e.g. because it leads to suffering or death and does not follow from exaggerated social expectations towards the individual, we should reflect on whether it ought to be treated as medical problem by asking:

3. Does medicine provide the most adequate methods of understanding X and its causes?
  At which level (e.g. molecular, mental, social, several levels combined) do main causes of X occur?
  Are there any alternative, non-medical and more appropriate ways of understanding X and its causes?
4. Does medicalizing X ensure the most effective and safest methods of solving it?
  Are there any alternative, non-medical and more effective ways to solve X or its causes?
  Does medicalizing X do less harm than good?

The results of using the above guiding questions to evaluate selected examples of medicalization are presented in the Table above (Table 2).

**Table 2:** Exemplary use of four guiding questions—own elaboration

Medicalized X	Question no.			
	1	2	3	4
Myocardial infarction	√	√	√	√
Cancer	√	√	√	√
Poliomyelitis	√	√	√	√
Schizophrenia	√	√	?	√
Alcoholism	√	√	–	√
Anorexia	√	√	–	√
Male-pattern hair loss (MPHL)	√	–	√	–
Prolonged grief disorder (PGD)	√	–	–	–
Asymmetric labia as an indication for labiaplasty	?	–	–	–
Mild attention deficit hyperactivity disorder (ADHD)	?	–	–	–
Mild restless legs syndrome (RLS)	?	–	–	–
Hypoactive sexual desire disorder (HSDD)	?	–	–	–
Homosexuality	–	–	–	–

## Discussion

Let’s now analyse all of four guiding questions presented above, as none of them is unproblematic or self-evident. Some of the examples from the Table 2, will be used as illustration of those complexities.

It is the first question that definitely needs clarification. There is probably an endless number of X’s significantly increasing the risk of considerable physical or mental discomfort, suffering, impairments or death. The pragmatic approach proposed in this paper, however, was not meant to refer to all problems, but only to those which have begun or have already been medicalized—therefore defined by medical terms or solved using medical tools. This is why such life-threatening phenomena as war, poverty, pollution or car accidents were not included in Table 2. Although car accidents are taken into account in the World Health Organisation leading causes of death statistics (WHO 2017), and air pollution is often discussed in the context of its health consequences (WHO 2013), it seems reasonable to claim that they are not (yet?) seen as medical conditions.

However, the number of medicalized X’s is still large and growing (Conrad 2007, p. 112). What criteria can be taken as a basis of judgement that a given phenomenon has been rightly recognised as a problem at all? There is a number of tools that can be helpful in order to assess whether or not X causes or significantly increases the risk of considerable physical or mental discomfort, suffering, impairments or death. One of them is the concept of *disability weight* used by the World Health Organization to reflect and compare severity of diseases (WHO 2004) and related to it *disability-adjusted life year* (DALY) as a measure of global disease burden:

One DALY can be thought of as one lost year of “healthy” life, and the burden of disease can be thought of as a measurement of the gap between the current health status and an ideal situation where everyone lives into old age, free of disease and disability. DALYs for a disease or injury cause are calculated as the sum of the years of life lost (YLL) due to premature mortality in the population and the years lost due to disability (YLD) for incident cases of the disease or injury (WHO, WTO, WIPO 2013, p. 26).

Statistical data provided by the World Health Organization can be an important reference point while looking for the answer to the first guiding question, although there are three main objections that can be raised against such approach. First, WHO’s calculations refer to the concept of health, which has been criticized in this article—nevertheless the core element in measuring disability weight and DALY is premature death, which seems to be far less ambiguous term than the notion of health itself. Thanks to the evidence-based medicine and population studies, we can establish which medicalized X’s are statistically more lethal.

The second objection is more fundamental. It undermines the efforts to quantify and measure immeasurable experience of illness or to portray human suffering with a single statistical model. The limitations of statistical approach are particularly visible when it comes to assessing mental discomfort. This is why, although scientific data about disability and mortality caused by certain X should be taken into consideration while answering the first question, the answer cannot be based only upon them. Personal experiences and subjective testimonies of suffering given by people experiencing X are the second, less quantifiable, but equally important source of information.

Having included personal experiences, we can also face the third objection which rightly points out that the data provided by WHO would be irrelevant whenever the debate about medicalization concerns X that was not recognised as a disease before—in such cases there would be simply no statistical or medical data about this condition.

Let’s consider as an example the male-pattern hair loss (MPHL). Thanks to the evidence based-medicine we can undoubtedly tell that this indisposition is not lethal and it does not cause any physical pain—as such it is far less severe than poliomyelitis or cancer. What is more, at a certain age, MPHL is so common that one could even call it a normal or typical condition. In some cases, however, it may cause significant mental distress (Cash 1992)—consequently, according to the pragmatic framework, it is enough to be seen as a problem.

The second guiding question in the pragmatic framework seems to be much more difficult to answer. Based on what criteria is it possible to establish whether or not recognising X as a problem results from unfounded, exaggerated social expectations? How to draw boundaries between proper and inappropriate limitations of diversity of individuals? The whole process of socialization, though, requires from an individual to adjust to social norms. Despite the fact that there are no precise criteria on that matter, two helpful hints can be proposed.

The first one refers to the history of medicine, social prejudices and discrimination. We know from the past that socially constructed norms can be marked by racism or gender bias. The sociological works on biopolitics (Lemke 2011) may be an useful source of examples, showing how human body and behaviour can be excessively disciplined in the name of norm or health. For instance, current studies on that matter demonstrate that the development of genetics, combined with modern social norms, may strengthen the notion of personal responsibility for health, which may possibly lead to new medicalization practices, performed in the name of determining susceptibility or in the name of prevention (Rose 2007b, pp. 40, 92–93, 241–243; Vogt et al. 2016). Thanks to the studies on social norms and oppression we should know which cases of medicalization are potentially suspicious, although some completely new forms of social constraints may also emerge.

The second hint refers to commercial interests involved in progressive medicalization. Popular beliefs about what is normal, what is treatable and what is desirable can be to a large degree influenced by media discourse and advertising. This phenomenon is now called disease mongering or disease branding (Payer 1992). As Carl Elliott explained:

To brand a disease is to shape its public perception in order to make it more palatable to patients. This is usually done by telling people that the disease is taken seriously by doctors, that it is far more common than they ever realized, and that having it is nothing to be ashamed of (Elliott 2010, p. 120).

One of the declared goals of drug advertising and health awareness campaigns is to foster the de-tabooisation of a given disease. As it was shown in the Table 1, in general, de-tabooisation of disease is seen as a positive aspect of medicalization. This positive characteristic, though, can be a double edged sword—especially when de-tabooisation enables creating a “fashion” for a given disease or expanding demand for specific pharmaceuticals.

According to some authors, the male-pattern hair loss (MPHL) is a perfect example of such a promoted, boosted condition, because it started to be seen as a problem after a series of advertisements and news releases alarming about negative psychological effects of baldness, proven in the studies funded by one of the drug companies (Moynihan et al. 2002, p. 887). Taking into account the fact that male-pattern hair loss at a certain age is a typical condition, and that it could be promoted as a cause of depression by pharmaceutical industry, one may argue that medicalization of MPHL is a result of unfounded, exaggerated social expectations.

The answer to the second guiding question, however, will often be contentious. Today, individuals use medical means to better fit into social imagery about what normal and fulfilling womanhood (the case of aesthetic surgery), manhood (the case of erectile dysfunction) or childhood (the case of ADHD) is. The challenge here is to distinguish between authentic needs and those demands which were created by advertising or oppressive social expectations (grounded, e.g., in gender bias). Although in many cases it might be impossible to make a clear cut^7^ distinction between those options, some practices, like psychological consultation before a given medical procedure, may help the patient to better understand his or her own motivations. There will probably always be, however, a tension between the idea of authenticity as self-acceptance and authenticity as self-creation (Parens 2005). When it comes to baldness, but also aging in general, some would prefer to accept it as an inevitable part of life, while others would look for any means to defeat it.

The third and fourth questions in the pragmatic framework seem to be only a little less problematic than the second one. In order to know if medicine provides the most adequate methods of understanding X and its causes we need to refer to the scientific knowledge about this phenomenon, state of the body, behaviour or sensation. X may be determined by some genetic or hormonal factors, it may be caused by viruses, fungi, bacteria or parasites, it may be a problem generated by organ dysfunction or injury. Nonetheless, X may be also caused by personal experience (e.g. traumatic event), poor living conditions, stress, lifestyle, interpersonal relations, or diet. Causes of X may be, therefore, situated at the molecular, physiological, psychological or social level, yet obviously those levels are often interrelated and combined. One might even argue that—as there are complex philosophical issues of relationships between body and mind or nature and culture—we cannot be ever sure where the deepest source of our problems lies. The pragmatic approach assumes, however, that we have to base our practices on the imperfect knowledge we possess.

What is important in the context of the third question is that modern biomedicine is reductionist (Rose 2007b, p. 11), yet this reductionism is more adequate in some than in other cases. Whenever there is no clearly identified pathogen or other physiological cause of X, some other disciplines than medicine should be also used to try to better understand a given phenomenon.

Let us take alcoholism and anorexia as the examples. Both those conditions significantly increase the risk of suffering or death. Considering them as problems does not seem to result from unfounded, exaggerated social expectations. Yet medicine does not provide the most adequate methods of grasping them. They can be understood not only as psychiatric disorders, but also personal, psychological and social challenges. There are important institutional and cultural factors influencing prevalence of alcoholism and anorexia. In the case of addiction, what matters is the attitude toward using drugs in a given society, access to them, but also unemployment or life opportunities. In the case of anorexia, gender stereotypes and canons of beauty are not without significance. Those other-than-medical levels of explaining alcoholism and anorexia are something more than just factors that increase their occurrence. They convey an important message about what those complex problems are per se.

In this respect, alcoholism and anorexia are significantly different than diarrhoeal disease which still is the second main cause of death in low-income countries (WHO 2017). Although what makes it so murderous is the political and economic situation—tragic living and sanitary conditions in the poorest countries, lack of clean water and basic medicines—what directly causes this illness often is a simple pathogen, such as rotavirus. In other words, poverty is the main reason why diarrhoeal disease remains lethal in many countries, but as a condition in itself it can be fully described at physiological level.

Adequate understanding of X and its causes is significant in establishing the most effective and safest methods of solving it, although it is not always necessary. This is a reason why, in the pragmatic framework, the question number three and four are separated. Sometimes, even when the exact causes of a given disease are unknown, medicine can provide better means of coping with it than any other alternatives might. This description seems to fit the case of schizophrenia treated with psychotropic drugs. And, to the contrary, sometimes, even when the medical causes of a given illness are well known, medicine alone cannot eliminate it. It seems to be the case of diarrhoea in the low-income countries. Vaccines against retroviruses are needed, but the disease can be also effectively defeated with political, not medical, means—namely by improving sanitary conditions, minimising extreme poverty or providing better education.

The last, fourth question, is devoted to exactly that issue. It may be seen as the most pragmatic question in the pragmatic framework, because it is not about what X truly is, but which solutions of the problem work best. At this stage of reflection there is a place for the risk–benefit ratio of medicalizing X. Whenever there are other, non-medical, more effective or safer ways to cope with it, they should be seriously considered.

Let us return to the example of male-pattern hair loss (MPHL). Even if one would recognise it as an authentic problem, furthermore, well scientifically explained at the hormonal level—still, it doesn’t imply that medicine might provide the best tools to cope with it. Some medications used to treat baldness, for instance, were accused of causing sexual dysfunctions (Irwig 2012). Every drug, of course, may cause some adverse side effects but, in the context of mild diseases, those side effects can shape the risk–benefit ratio in such a way that it will be more risky to take medication than to accept a given indisposition.

Naturally, if medicalization of X doesn’t provide any helpful means to cope with this problem, it does not imply that X may not be seen as a medical issue. It still can be understood as such if medicine enables us to better understand it (and if that is the case, than there is also hope we may discover how to cure it in the future). Nevertheless, if X can be neither explained nor healed by the tools of medicine, it may be over-medicalized.

In trying to estimate this risk–benefit ratio of medicalizing X, we can refer to the scientific data about efficacy and safety of a specific drug or medical procedure used to treat it, optimally gained from a source unbiased by commercial interests, such as Cochrane Library.^8^ What is more, to make proper estimations, we should already have answers for the first two questions in the pragmatic framework. What is the benefit of eliminating X which is completely harmless or is seen as a problem only because of exaggerated social expectations?

In order to better understand the pragmatic framework proposed, let’s conduct a little thought experiment. We may assume that a new psychiatric disorder—called highway menace syndrome—was described in DSM-X. People who were diagnosed with such a syndrome (mainly psychological tendency to aggressive driving) are statistically more disposed to cause car accidents. How should we assess such a case of medicalization using the four guiding questions?

We would probably acknowledge that highway menace is a real, authentic problem (even though the new syndrome has been strongly promoted by a pharmaceutical company which sells a psychotropic drug for this condition). How would we answer, however, the third and fourth question? There are at least two options. The first one seems to be much more probable: we would maintain that aggressive driving can be both explained and effectively managed without any tools of medicine. We would argue that aggressive driving is not fully determined by psychological tendencies—it is also a matter of free will of a driver, driving culture of a given society etc. What is more, car crashes could be prevented by traffic regulations, effective law enforcement, better education, social campaigns or perhaps even by replacing drivers with autonomous cars. We have alternative ways of understanding and coping with aggressive driving—so why would we have to use the new drug for highway menace syndrome (especially knowing that it may cause some serious side effects)? Taking that all into account we would assess this case of medicalization negatively.

The second option is far less probable but also interesting. Let’s assume that a drug for the highway menace syndrome—called a super-driver pill—is perfectly safe for health and really effective. For a given period of time it eradicates the driver’s tendency to unreasonable, risky driving behaviors, additionally, it improves concentration, eyesight, reflex and decreases sleepiness. What is more, the pill has proved to be much more effective in preventing car crashes than any other means. Should people who were diagnosed with highway menace syndrome be encouraged to take it? Would it be still an example of over-medicalization?

Of course, here we would enter a separate ethical debate about using tools of medicine for cognitive or moral enhancement. There are strong arguments for and against human enhancement which have nothing to do with the problem of medicalization—and there is no room in the article to explore that issue. However, it should be noted that the pragmatic framework itself does not definitely exclude using tools of medicine for human enhancement. The medicalization of highway menace syndrome in the second scenario would be less questionable than in the first, more probable one. It implies that, in some cases, according to pragmatic framework, we could consider using tools of medicine to cope with problems which may not be recognized as diseases.

## Conclusion

Although in many cases the answers to the four questions proposed above will not be obvious, there is no doubt that medicalization of certain issues proves to be much more contentious than medicalization of others.^9^ The more question marks or negative answers are given to the four guiding questions, the more objections can be raised as to the medicalization of a particular phenomenon. Furthermore, in such case, the risk of the aforementioned adverse consequences increases as well, among them: overdiagnosis (Hofmann 2016), overprescription, adverse side effects caused by medication, iatrogenic diseases, suboptimal expenditure and waste of public or private money, stigmatisation and inadequate reactions to a problem (e.g. attempts to address a social or political problem at the level of molecular intervention in individuals’ bodies).

The aim of the above considerations regarding over-medicalization has not been, however, to justify prohibition of taking medication when their adequacy might be disputable. The point, therefore, is not to refuse the right to take antidepressants to those unable to overcome their grief, or to prohibit women from undergoing plastic surgery just because they wish for their own bodies to conform to socially constructed canons of beauty. Controversies aroused by medicalization of a given phenomenon could be seen, however, as a warning sign.

The pragmatic approach to assessing medicalization assumes that we are to seek the most adequate, effective and safest ways of addressing specific problems, which is by definition highly sensitive to the context and personal preferences of individuals. The four guiding questions proposed in this article should contribute to a sociological and bioethical reflection on the boundaries of medicine. They should also provide a point of reference for selecting tools to be used to solve each problem. In many cases, the tools offered by medicine may not be optimal.

## Footnotes

1 Although there is no room in the article for in-depth ontological or epistemic considerations, it should be noted that whenever I use such words as “reality”, “causes”, “objective factors”, “undoubtedly” or “in fact”—they should be interpreted in the context of pragmatic theory of truth.

2 This is also one of the reasons why I disagree with Schramme’s (2007, p. 12) claim that naturalistic concept of health can serve as a “gatekeeper against medicalization”.

3 Compare differences between norms of blood pressure in The Seventh Joint National Committee on Prevention, Detection, Evaluation and Treatment of High Blood Pressure (JNC 8) and guidelines of the European Society of Hypertension.

4 Although the purpose of this article is not to enter into the dispute between the normativist and naturalist concepts of health—it should be noted that the following considerations fit the former better. Recognizing that health and disease are value-laden concepts, however, doesn’t imply that they are fully socially constructed.

5 I refer to Ercan Avci’s presentation at the 31st European Conference on Philosophy Of Medicine And Healthcare (ESPMH) entitled *Ethical concerns resulting from the advances in life-sustaining technologies: the transformation of the “*ars moriendi” *to* “medicalized death”.

6 Although the goal of this article is not to define the disease, the pragmatic framework I propose is to a certain degree consistent with the Peter Schwartz claim that “definitions of “disease” should not be seen as traditional conceptual analyses—that is, claims about the term’s current meaning or criteria for application—but instead as proposals about how to define and use the term in the future” (Schwartz 2017, p. 485). My proposition can be also seen as an addition to de Vreese’s (2017) pragmatic approach to the notion of disease.

7 Paraphrasing Rogers and Walker (2017, p. 421) it could be said that medicalization—just as disease—is “a vague concept” and “a matter of degree”. If we imagine the phenomenon of medicalization on a scale—as a continuum from “too little” to “too much” medicine—on one end of such a scale there would be underdiagnosis, somewhere in the middle there would be well-founded medicalization while over-medicalization (with overdiagnosis as one of its effects) would be located on the other end of the scale. Inevitably, there would be also “borderline cases that are not clearly” well-founded medicalization or over-medicalization (2017, p. 421).

8 Useful methods of assessing patient benefits and harms are also described in *Guidance for Modifying the Definition of Diseases* (Doust et al. 2017a, b).

9 Cancer or heart attack (and other examples of “well-founded medicalization” in Table 2) have been medicalized for such a long time, that it might be hard to imagine that they are treated and understood not as medical problems. Ethnographic studies of different traditional communities show, however, that biomedical explanations of such phenomena can co-occur with explanations of other types (Tenzel 1970; Yeo et al. 2005; Prior 2009). In some areas of the world, western medicine has to compete with traditional (e.g. religious, spiritual, shamanic) ways of coping with such phenomena as cancer. Illnesses may be still understood as a result of divine punishment, evil eye, witchcraft, bad spirits or karma—which may delay medical treatment. The four guiding questions show that the medicalization of cancer is justified because medicine can explain and cope with it better than any other social practice.
